# Introduction: Laboratory automation at Schering-Plough—increased productivity today and foundation for the future

**DOI:** 10.1155/S146392469500006X

**Published:** 1995

**Authors:** Francis H. Zenie

**Affiliations:** Zymark Corporation Zymark Center Hopkinton MA 01748 USA

## Abstract

The following is the introductory presentation to the 1994 ISLAR meeting held in Boston from 16 to 19 October 1994. The Editor is again grateful to the organizer, the Zymark Corporation, for permission to publish the papers in the Managing Laboratory Automation Session read at ISLAR.


Journal of Automatic Chemistry, Vol. 17, No. 2 (March–April 1995), pp. 41–45

### Introduction

### Laboratory automation at Schering-Plough—increased productivity today and foundation for the future

**Francis H. Zenie**
*Zymark Corporation, Zymark Center, Hopkinton, MA 01748, USA*

*The following is the introductory presentation to the 1994 ISLAR meeting held in Boston from 16 to 19 October 1994. The Editor is again grateful to the organizer, the Zymark Corporation, for permission to publish the papers in the Managing Laboratory Automation Session read at ISLAR.*

### Preface

ISLAR 1994 makes many years of hard work worthwhile and the future more exciting than ever. When we began Zymark, we stated our vision in our May 1991 introductory brochure—*Zymark—Dedicated to Increased Productivity in Chemistry and the Biotechnologies.* This statement preceded conceiving the laboratory robotics idea: 'Perhaps the most critical element limiting technological progress will become the availability and utilization of skilled people. The chemical and biological sciences have traditionally enjoyed an abundance of trained people, resulting in many labour intensive practices in their research laboratories. At Zymark, we believe it will become evident in the near future that this abundance is rapidly ending.'

We were correct in anticipating a productivity crisis, but incorrectly defined the cause. We saw robust and expanding chemical and pharmaceutical industries desperate for skilled people. Today's productivity crisis, however, is caused by increasing customer demands, competition and regulation.

We now have a choice: become depressed by this reality or challenged to create opportunities within it. Examining the causes of the productivity crisis, we find that it starts with us as consumers. We have become far more demanding consumers and our suppliers must continuously re-earn our business. Think about purchasing a new house or car or even small appliance: to compete and earn business, companies must create more value—better products and services at lower cost. This means jobs and work must change. Businesses must re-engineer their processes and we, as individuals, must become more valuable by renewing and exanding our skills.

We used to ask whether 'automation eliminates jobs'? This is the wrong question particularly for high value-added jobs. Senior executives in large companies are decisively reducing costs based on their perception of affordability. Senior managements are often measured by near-term financial performance and are responsible to their boards of directors who, in turn, are responsible to the shareholders. The largest, most powerful shareholders, are institutions such as pension funds, insurance companies and mutual funds. But, whose money is it? Ours, and we demand high returns on our investment.

We cannot predict the future and the *right* strategies are not clear. In fact strategies in your industries are changing and actually diverging. Only after three to five years, we will learn which managements made the best decisions. Once senior management adjusts their cost structure, they challenge middle management: 'find a way to do the necessary work within these financial constraints'.

I have commented at prior ISLARs about the changing role of laboratory management. We have virtually completed the first phase: the transition from technical expert to leadership and managerial duties. The next step is to become champions and leaders for increasing productivity and effectiveness. And, then, we must renew our commitment to innovation in order to regain control of the future and not just respond to it. But, we must do this while continuing to increase productivity. And technology, such as laboratory automation, is a tool to help people become more productive and innovative.

The following is a case history: it describes the 10-year evolution of laboratory automation at Schering-Plough which will demonstrate one company's commitment to preparation for the future.

Increasing cost containment pressures, worldwide competition, and government 'health care reform' initiatives demand increased productivity throughout today's pharmaceutical industry. To improve the critical analytical support required for new product development and ongoing quality control, leading pharmaceutical laboratories are re-engineering themselves to become more productive and to use valuable people more effectively. New methods and technologies are needed to further stimulate better laboratory support—fully compliant with current regulations and at lower cost.

Beginning as early pioneers, Schering-Plough has applied laboratory automation to dramatically increase their analytical and quality control productivity. Their early

© Zymark Corporation 1995

vision, commitment to improving productivity, belief in enriching career development and determined implementation provide important insights for other organizations in earlier phases of their laboratory automation programmes.

### Schering-Plough's beliefs

Schering-Plough, a leading research-based pharmaceutical company, has a rich tradition of introducing innovative products and maintaining a strong R&D pipeline. Schering's management recognizes the challenges ahead and they are committed to investing in R&D and in increasing the productivity of valuable R&D staff.

> 'The risks and challenges inherent in today's worldwide pharmaceutical business have never been greater. Conversely, the opportunities for Schering-Plough have never been more plentiful or full of promise.'
>
> Letter to Shareholders—Schering-Plough Corporation, 1992 Annual Report
>
> 'Our strategy for many years has been built upon a fundamental commitment to research. Only through investing in R&D will we discover and develop tomorrow's life-saving and life-enhancing pharmaceuticals. Only through perpetual innovation will Schering-Plough continue to grow and prosper.'
>
> Letter to Shareholders—Schering-Plough Corporation, 1993 Annual Report
>
> '**The quality of R&D depends on the quality of the resources.** There are a number of components to success in the pharmaceutical research process. The most important is our people. At Schering-Plough, we hire the best talent in our industry. Then we provide them with the technical resources they need to do their best work: the most advanced instrumentation; the latest computerized systems; modern lab facilities. Finally, we spend over $500 million on R&D, and have just completed a $300 million R&D facility.'
>
> Shering-Plough Research Institute—Employment Advertisement, New York Times, Sunday, August 29th, 1993.

### Laboratory automation—getting started

Following commercial introduction at the 1982 Pittsburgh Conference, laboratory robotics became quickly recognized as a powerful new technology for improving both laboratory productivity and the reproducibility of analytical results. Steady progress during the past 12 years has brought laboratory robotics technology and application know-how to its current capability and critical role in improving productivity for leading laboratories.

Not surprisingly, Schering-Plough adopted laboratory robotics technology early. Beginning in 1983, Schering Research introduced robotics systems for enzyme assays and Lowry protein assays. Schering's Physical and Analytical Chemistry R&D (PACRD) acquired their first robotic system in 1984 for tablet assays. Schering Quality Control began applying robotics in 1986 as part of an emerging commitment to lab automation. Automated tablet dissolution testing was QC's initial application—followed by automated metered dose inhaler assay. Today, Schering-Plough is a major user of a wide range of laboratory automation technology.

At Schering-Plough, laboratory automation includes automated sample preparation, automated analysis and automated data acquisition, processing and analysis. Each of these automation areas contribute important value and, combined into integrated laboratory automation, further increases the pay off.

Eugene McGonigle, Schering's Vice President of Physical and Analytical Chemistry R&D, in his keynote address at the 1992 ISLAR described the impact of laboratory automation on a typical gas chromatographic assay:

'Pre-Seventies: One chemist—5 Samples per Day
Seventies: One Chemist—10 Samples in $1\frac{1}{2}$ Days
Eighties: One Chemist—30 to 40 Samples per $1\frac{1}{2}$ Days'.

This modern Automation/Robotics/Computers, it is possible to generate twice the workload in half the time—*a fourfold productivity improvement.*

Analytical R&D and quality control functions have different, yet complimentary, objectives for laboratory automation. In analytical R&D, laboratory automation is used to increase productivity and improve sample turnaround for repetitive analyses supporting drug development. Analytical R&D must determine when, during the drug development process, to develop and utilize automated methods. Automating too early may not pay back because many projects are cancelled during early development stages and, for those that proceed, formulations and analytical methods frequently change. An equally important goal of analytical R&D is methods transfer to quality control laboratories for product release and stability analysis. Analytical R&D, consulting with the quality control staff, develops and validates manual-equivalent automated methods for subsequent, high volume use.

Supporting marketed products, quality control performs efficient and regulatory compliant analytical methods. In today's business environment, QC relies on laboratory automation to gain productivity by increasing analytical capacity with limited staff and space, while ensuring consistent use of validated methods and automating required documentation.

### Physical and analytical chemistry R&D

At Schering-Plough, Physical and Analytical Chemistry R&D (PACRD) provides analytical support throughout the pharmaceutical development process. Primary techniques include content uniformity, dissolution and stability indicating assays.

Pharmaceutical development proceeds in phases and analytical support is particularly difficult during the early phases, because:

- Many projects are terminated early in the development process.

- Early clinical trials involve few patients using preliminary formulations and dosage forms. Very often, each employ different analytical methods.
- Initial formulations are refined and additional strengths added or modified entirely as the development process proceeds through later stage, higher volume clinical trials.

Therefore, while many stability samples require testing, they are placed under stability at different times, making it difficult to combine or pool related analyses. The challenge in analytical R&D becomes *when* to introduce automated methods during the development process.

Continuing advancements in automation technology reduce the effort and time needed to adopt automated techniques for smaller sample loads and, thereby, enabling cost-effective automation to be introduced earlier in the development process. Zymark's PyTechnology, introduced in 1986, expanded the use of laboratory robotics at Schering-Plough. More recent developments, such as modular robotic workstations and powerful, Windows-based software with intuitive user interfaces and automatic sample scheduling are further lowering the barriers to automation. As the flexibility and ease of implementing laboratory automation increases, automation can be introduced earlier in the development process.

Schering's PACRD carefully evaluates each analysis for 'payback' return on the resources invested in developing the automated method. The example in table 1 illustrates an assessment for a solid dosage form, single component tablet or capsule assayed by HPLC. This demonstrates effective use of robotic applications when supported by people experienced in implementing laboratory automation technology. The incremental addition of nine days to program and validate the automation is a small part of the methods development project and the additional time is easily recovered even with moderate sample work loads.

Teaming vendor resources and internal laboratory expertise further facilitates the adoption of laboratory automation during pharmaceutical development. Internal laboratory staffs are more familiar with their products, regulatory and analytical needs and, therefore, internal expertise often leads to faster implementation and validation. For example, PACRD has decentralized internal teams that maintain an inventory of automation modules, configure systems quickly when needed, program the application and validate methods. Backing up these teams is Schering's R&D Engineering Department which provides additional engineering, programming and fabrication capabilities.

*Table 1.*

| Activity | Days required | % of total effort |
|---|---|---|
| Develop HPLC system & assay | 13 | 34 |
| Sample preparation | 3 | 7 |
| Validate manual method | 10 | 25 |
| Robotic programming | 3 | 7 |
| Robotic validation | 6 | 15 |
| Document methods | 5 | 12 |
| *Total* | 40 | 100 |

Full service system integration vendors, such as Zymark, bring proven products, know-how from similar applications, expertise in lab automation technology, custom product capabilities and a wide range of software knowledge including interfacing with other manufacturers products. Specific project needs determine the best mix of internal and vendor resources.

A recent project, automating content uniformity of a metered dose inhalation product, required development of new robotic modules and substantial empirical optimization of dose recovery approaches. The technically challenging project progressed slowly until a focused effort was contributed by the vendor (Zymark), Schering's PACRD Staff and Schering's R&D Engineering.

When asked, 'Hou would you approach a new robotics application, requiring method development and empirical optimization, in the future?', a Schering scientist replied:

> 'Involve Schering R&D Engineering early.
> Share manual procedures with the vendor—identify all critical issues learned from manual experience.
> Maintain open, frequent communication with vendor.
> Visit vendor for comprehensive pre-shipment review.
> Streamline installation process—prioritize issues and solve critical issues first.
> Schedule timely analytical support to ensure rapid progress during installation.
> Install and test systematically, expect problems and test subsystems, do not run full protocol until success is expected.
> Assess future quality control needs and objectives'.

Based on our experience at Zymark, we agree'.

### Quality control

Supporting a comprehensive line of marketed products and a flow of new products, rapidly increases the analytical demands of pharmaceutical quality control. Established methods must be run as efficiently as possible so skilled resources have time to resolve problems quickly and prepare for new products. Schering-Plough's Quality Control Department relies on laboratory automation to increase analytical capacity, ensure excellent reproducibility and provide documentation and audit trails.

From the R&D viewpoint, McGonigle points out [1]: 'Methods transfer is a stringent requirement for analytical research and development laboratories. Our main charter, once stability has been demonstrated and specifications developed, is to transfer analytical methods, developed in our laboratories, to other potential sites who will be required to use these methods to release new products and continue marketed product stability programs. Method transfer requires a strong interface with Quality Control Staff.

As an early user of laboratory robotics, Schering took advantage of technological advancements such as Zymark's PyTechnology for system flexibility, System V controller for computer compatibilty and AccuTrack positioning for

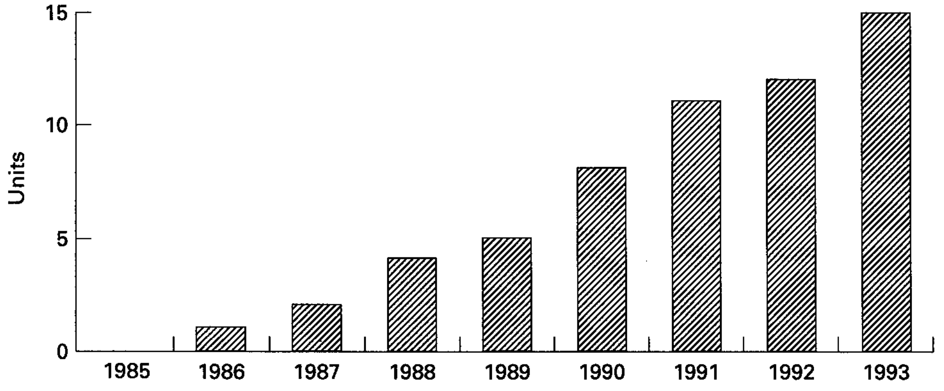

*Figure 1.*

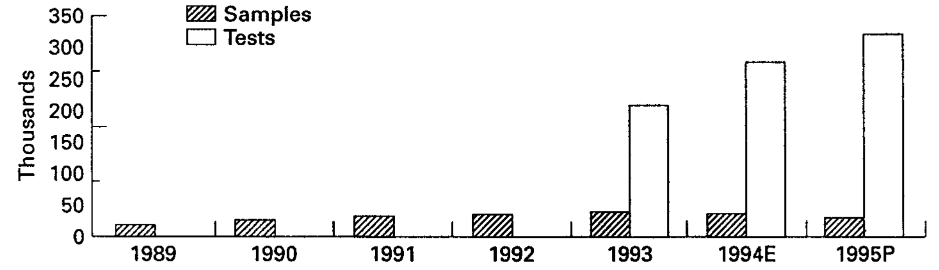

*Figure 2.*

*Table 2.*

| Year | Samples tested | Permanent lab staff | Number of tests | Annual samples/staff | Average tests per sample |
|---|---|---|---|---|---|
| 1989 | 15 500 | 79 | | 190 | |
| 1990 | 22 200 | 65 | | 342 | |
| 1991 | 30 200 | 77 | | 392 | |
| 1992 | 32 000 | 76 | | 421 | |
| 1993 | 37 000 | 91 | 208 200 | | 5.5 |
| 1994 est. | 34 300 | | 276 500 | | 8.1 |
| 1995 proj. | 30 900 | | 324 500 | | 10.5 |

improved system reliability. They became early adopters of automated workstations such as Zymark's BenchMate and BenchMate Tablet Processing Workstations. To further ensure success. Schering QC maintains a strong commitment to training their staff for laboratory automation.

Figure 1 illustrates the steady addition of laboratory automation systems at Schering Quality Control [2]. These systems include robotics systems, automated workstations, semi-automated dissolution systems and automated spectrophotometers. This ongoing commitment has paid-off in ever-increasing productivity.

Figure 2 and table 2 illustrates rapid sample growth through 1993 with virtually unchanged laboratory staff. Schering's total QC staff increased significantly during this period to support non-laboratory requirements. Now, with projections of level or slightly lower sample workloads, analytical demands continue to increase as regulatory compliance requires most tests per sample. Automation, therefore, is becoming more critical to support ever increasing analytical requirements.

As common assays such as content uniformity, composite assays and dissolution testing are routinely automated, automation vendors and pharmaceutical laboratories must continue to develop techniques for newer, more demanding assays. Table 3 illustrates additional tests being proposed for inhaler delivered drugs. The projected

*Table 3. Analytical testing of metered dose inhalers.*

| Current testing | Proposed testing |
|---|---|
| Description | Description |
| Identification (IR/HPLC) | Identification (IR/HPLC) |
| Leak test | Leak test |
| Water content | Water content |
| Particle size (microscope) | Particle size (microscope) |
| Weight per metered dose | Weight per metered dose |
| Assay: metered dose | Assay: metered dose (Beg. & end)* |
| | Total can assay |
| | Chromatographic impurities |
| | Particle size (cascade impactor)* |
| Applied time per batch = 20 h | Application time per batch = 75 h |

* *Note*: Indicates significant impact if not automated.

*Table 4. Manual versus automated testing—Applied time per batch.*

| Product | Manual | Automated* | Time saving (h per batch) | Annual time saving |
|---|---|---|---|---|
| Inhaler product 1 | 12 | 6 | 6 | 1900 |
| Inhaler product 2 | 12 | 5 | 7 | 800 |
| Inhaler product 3 | 12 | 5 | 7 | 300 |
| Tablet product 1 | 16 | 8 | 8 | 1200 |
| Tablet product 2 | 8 | 4 | 4 | 1200 |
| Tablet product 3 | 6 | 3·5 | 2·5 | 150 |
| Total annual time savings | | | | 5500 h |

*Note: Time includes system start-up/clean-up and report preparation.

increase in applied time per batch illustrates that more demanding analytical controls must be met by advanced automation techniques or significantly higher laboratory staffing.

In quality control, laboratory automation flexibility is required for high utilization of automated systems in order to ensure high return on financial investment and efficient space utilization. Table 4 illustrates how Schering QC automates both high and low volume tests to accumulate large annual time savings.

### Insights

We are still early in the evolution of laboratory automation technology, but effectively applied, laboratory automation pays off. Working together, vendors and users of laboratory automation can ensure near term payback and build a foundation for the future. Specific challenges include:

1. Lower the 'Automation Threshold'. That is, reduce the effort required to automate new methods so that automation can be introduced earlier in the drug development process and be easily applied to multiple methods in quality control. This requires every-improving technology and know-how from automation vendors and knowledge centres within end-user organizations.
2. Expand laboratory automation capabilities. Identify critical laboratory automation needs that require new technology or know-how. Develop these applications as collaborative projects with shared responsibilities and investment between vendors, end-users and internal automation staff.
3. Make automated methods more reliable and rugged. Payback comes from dependable operation.

'Unified Methods', made possible by methods transfer between laboratories, are essential to regulatory compliance with high productivity. Wherever possible, therefore, laboratories should concurrently develop equivalent manual and automated methods.

### Looking forward

Scherling-Plough continues as an active participant in the laboratory automation revolution. They have build a solid base of successful applications and formed a core of skilled automation specialists. Senior management also recognizes the capability and potential of laboratory automation.

Having achieved departmental success, Schering-Plough is raising their sights to interdepartmental considerations of methods transfer leading to unified methods. Most important, Schering now has a foundation of people and technology to meet the ever increasing challenges ahead.

### Acknowledgments

I would like to thank the following Schering-Plough staff members for their insights, assistance and cordiality: in PACRD—Eugene McGonigle, Don Chambers, Ed Mularz, Larry Lorenz, Doreen Schwanenflugel and Bob Foester; in Quality Control—Maurice Green, Jill Porcek, Kathy Ku, Tatyana Metter and Kathleen Mays.

### References

1. McGonigle, E. J., In *Proceedings for International Symposium on Laboratory Automation and Robotics* (1992), pp. 1–16.
2. Greene, M., *Automated Pharmaceutical Analysis*, presented at the Executive Forum Breakfast Meeting, 9 June 1994.

